# The COVID-19 pandemic: Is it a turning point for E-health? CON

**DOI:** 10.1192/j.eurpsy.2021.36

**Published:** 2021-08-13

**Authors:** H.-J. Möller

**Affiliations:** Psychiatric Hospital, Ludwig-Maximilians-University Munich, Munich, Germany

## Abstract

The Covid-19 Pandemia is not a turning point for e-health It might seem meaningful that this e-health approach will also continue in mental health care after the Covid-19, given the obvious pragmatic advantages. But several issues could at least delimitate a future use of e-health technology in psychiatry/psychotherapy: Patient with severe depression and suicidality seem not to be the right target group Same with other serious mental disorders First contact patients (depending among others on their personality characteristics), often have problems to open themselves at phone or video contacts In Germany (and probably other European countries?) a relative high percentage of psychiatrists/psychotherapists are reluctant or at least ambivalent against video based interventions A quite high percentage of psychiatrists/psychotherapists in Germany believe that a relevant proportion of their patients might not accept this very technical approach of doctor-patient-interaction. Currently in Germany, probably also in some other European countries, complain that some legal and billing issues are not fully solved. Some of these problems could decrease in the future and insofar it is difficult to make a valid prediction of the place of e-health technology in psychiatry/psychotherapy. Beside this one point seems of great importance to me: the current e-health success, driven by the Covid-19 Pandemia, should not be the direction of more cost saving psychiatry/psychotherapy, generally neglecting the need for personal interaction between patients and therapists.

**Disclosure:**

No significant relationships.

